# Systolic blood pressure variability in patients with early severe sepsis or septic shock: a prospective cohort study

**DOI:** 10.1186/s12871-017-0377-4

**Published:** 2017-06-17

**Authors:** Yi Tang, Jeff Sorenson, Michael Lanspa, Colin K. Grissom, V.J. Mathews, Samuel M. Brown

**Affiliations:** 1 Pulmonary and Critical Care, Intermountain Medical Center, 5121 Cottonwood St, Murray, UT 84107 USA; 2 Pulmonary and Critical Care, University of Utah School of Medicine, 30 N 1900 E, Salt Lake City, UT 84132 USA; 3 Electrical and Computer Engineering, University of Utah, 50 Central Campus Dr #2110, Salt Lake City, UT 84112 USA; 4 Shock Trauma Intensive Care Unit, 5121 South Cottonwood Street, Murray, UT 84107 USA

**Keywords:** Sepsis, Shock, Physiological variability, Arterial blood pressure

## Abstract

**Background:**

Severe sepsis and septic shock are often lethal syndromes, in which the autonomic nervous system may fail to maintain adequate blood pressure. Heart rate variability has been associated with outcomes in sepsis. Whether systolic blood pressure (SBP) variability is associated with clinical outcomes in septic patients is unknown. The propose of this study is to determine whether variability in SBP correlates with vasopressor independence and mortality among septic patients.

**Methods:**

We prospectively studied patients with severe sepsis or septic shock, admitted to an intensive care unit (ICU) with an arterial catheter. We analyzed SBP variability on the first 5-min window immediately following ICU admission. We performed principal component analysis of multidimensional complexity, and used the first principal component (PC_1_) as input for Firth logistic regression, controlling for mean systolic pressure (SBP) in the primary analyses, and Acute Physiology and Chronic Health Evaluation (APACHE) II score or NEE dose in the ancillary analyses. Prespecified outcomes were vasopressor independence at 24 h (primary), and 28-day mortality (secondary).

**Results:**

We studied 51 patients, 51% of whom achieved vasopressor independence at 24 h. Ten percent died at 28 days. PC_1_ represented 26% of the variance in complexity measures. PC_1_ was not associated with vasopressor independence on Firth logistic regression (OR 1.04; 95% CI: 0.93–1.16; *p* = 0.54), but was associated with 28-day mortality (OR 1.16, 95% CI: 1.01–1.35, *p* = 0.040).

**Conclusions:**

Early SBP variability appears to be associated with 28-day mortality in patients with severe sepsis and septic shock.

**Electronic supplementary material:**

The online version of this article (doi:10.1186/s12871-017-0377-4) contains supplementary material, which is available to authorized users.

## Background

Severe sepsis and septic shock are life-threatening manifestations of severe infection, and afflict 750,000 patients annually in the USA with an associated mortality of 25–50%. [1, 2] While current consensus emphasizes early interventions to control sepsis, [3, 4] which intermediate endpoints and predictors are most useful for guiding interventions is not clear; nor are early markers of sepsis severity well established.

Connections between the parasympathetic nervous system and inflammation suggest that autonomic nervous system function and inflammation may be interdependent. [5] Furthermore, physiological systems exhibit nonlinear patterns of complexity, including fractal self-similarity across time scales. [6, 7] Early studies documenting an association between complexity in autonomic nervous system control suggest that such complexity may be a window on hemodynamic disarray. Whether such complexity measures are relevant in sepsis is unknown. Most of the work on cardiovascular complexity to date has focused on patterns of heart rate variability (HRV), which measures changes in the number of milliseconds between successive heartbeats. Less work has focused on the variability of arterial blood pressure. We therefore sought to define the association between arterial blood pressure (specifically, systolic blood pressure) variability and early outcomes in sepsis.

## Methods

### Setting

We studied patients admitted to one of two intensive care units (ICUs), the 24-bed Shock Trauma ICU, and the 12-bed Respiratory ICU, at Intermountain Medical Center, a 452-bed tertiary-care, academic hospital in Murray, Utah, USA. This study was approved by Intermountain’s Institutional Review Board (#1020798) with a waiver of informed consent.

### Patients

We prospectively identified adult (>15 years of age) patients with severe sepsis or septic shock at time of admission (as defined in then-current consensus guidelines [8]) to study ICUs from June 2012 to July 2013. We excluded pregnant patients, patients with admission Do Not Resuscitate / Do Not Intubate orders, patients without an arterial catheter placed for routine clinical monitoring, and patients who were not in sinus rhythm. For patients who had multiple ICU admissions during the study period, we only included the first time they were admitted to a study ICU with sepsis during the study period. Because new sepsis guidelines (SEPSIS-3 [9]) were published after the study was completed, we performed a secondary analysis to determine how many patients met SEPSIS-3 criteria.

### Clinical data

We calculated admission Acute Physiology and Chronic Health Evaluation II (APACHE II) [10] and Sequential Organ Function Assessment (SOFA) [11] scores in all study patients. Infusion rates of vasopressors (norepinephrine, epinephrine, dopamine, phenylephrine, and vasopressin) are automatically uploaded in real-time to the hospital Electronic Medical Record (EMR) as part of routine clinical care. We analyzed all vasopressors administered during the first 6 h after ICU admission, converting them to norepinephrine equivalent dosages according to standard equivalencies [12].

### Physiological data acquisition and processing

We sampled blood pressure data from bedside Philips Intellivue monitors using the Research Data Export (RDE) functionality. RDE provides 125-Hz digitized tracings of arterial blood pressure. We used the first 5 min of ABP data available for each patient. We identified the systolic peak for each heartbeat using a simple filtering technique to remove electrical mains noise and capture the first value that achieved the relevant local maximum, using a custom-written algorithm based on a hill climbing method and local maximum comparison. We manually validated this algorithm on random samples of patient data to confirm accuracy. The time series of systolic blood pressure (SBP) values was the fundamental blood pressure measurement for the purposes of this study. We excluded patients with inadequate quality tracings, based on visual inspection of the tracings.

### Clinical outcomes

Our pre-specified primary outcome was vasopressor independence at 24 h after ICU admission, an outcome we have studied previously and felt represented successful early resuscitation of sepsis. [13–15] To meet criteria for vasopressor independence at 24 h, a patient had to be alive and liberated from vasopressor therapy from 24 through 48 h after ICU admission. Our prespecified secondary outcome was all-cause 28-day mortality, which we determined from the Intermountain Death Record, which incorporates data from Utah state vital statistics.

### Statistical methods

We used Continuous Individual Multiorgan Variability Analysis (CIMVA™; Dynamic Analysis Laboratory, Ottawa) software to generate a full profile of complexity measures (including time-, frequency-, and complexity-domain metrics) for the first 5 min of measured SBP. We chose 5 min because we were interested in a complexity measure that could be applied quickly under clinical conditions. We then simplified those complexity metrics into their first principal component, using principal components analysis (PCA). Principal component analysis transforms a set of observations into a set of orthogonal values of linearly uncorrelated variables, defined such that the first principal component (PC_1_) has the largest possible variance.

We used Firth logistic regression, a bias-reduction modification of the maximum-likelihood approach useful for datasets with sparse outcomes, to evaluate the association between the first principal component (PC_1_) of SBP complexity and the primary and secondary outcomes. We built two separate models of outcome, one for the primary outcome of vasopressor independence at 24 h, the other for the secondary outcome of 28-day mortality. We included average SBP during the 5-min study window as a covariate in the regression, using variance inflation to assess for collinearity. For building our models, we started with a bivariate analysis incorporating average SBP. We tested model calibration with the Hosmer-Lemeshow goodness-of-fit (GOF) test, and discrimination by bootstrapping estimates of receiver operating characteristic areas under curve (AUC ROC). In sensitivity analyses, we estimated whether APACHE II and vasopressor infusion rate were important covariates and performed univariate regression. We controlled for the initial vasopressor infusion rate in a sensitivity analysis to improve the face validity of our findings, given the probable association between initial use (and dose) of vasopressors and vasopressor independence at 24 h. In a post hoc exploratory analysis we used exact logistic regression instead of Firth logistic regression (see Additional file 1). In another post hoc exploratory analysis, we inspected the association (using Firth logistic regression) of the coefficient of variation of systolic blood pressure with the first principal component and clinical outcomes, in order to understand whether simple variation in systolic blood pressure, as may be seen with hypovolemia, was driving the results. Statistical analysis and hypothesis testing was performed within the R statistical package (version 2.12) [16].

## Results

Of 75 patients with severe sepsis or septic shock with an indwelling arterial catheter, we studied the 51 (68%) patients with adequate quality arterial blood pressure waveforms. Table 1 depicts patient demographics and measures of disease severity. Patients had a median age of 60 years; half were female. The median APACHE II score was 27. Abdominal sepsis and pneumonia predominated among the causes of sepsis (Table 2), each representing a quarter of patients. All patients met SEPSIS-3 criteria for sepsis, while 27 (53%) met SEPSIS-3 criteria for septic shock; 12 (24%) met criteria for sepsis-induced hypotension (i.e., they required vasopressors but had a normal lactate).

**Table 1:** Patient characteristics

Characteristic	Central tendency
Total number of patients	51
Age, years	60 (47–70)
Female sex	27 (52%)
Admission APACHE II score, points	26.5 (18.5–34.3)
28-day mortality	5 (9.6%)
Vasopressor-free days at 28 days	23 (22–26)
Admission SOFA score, points	10 (7–12)
Admission systolic blood pressure	99 (87–109)

Descriptive statistics organized by diagnostic group. Data reported as mean (SD), median (25%–75% percentile), or count (percentage of total)

**Table 2:** Sources of sepsis

Sources of sepsis	Number of Patients
Abdominal	13 (25.5%)
Bacteremia	2 (3.9%)
Endocarditis	1 (2%)
Pneumonia	13 (25.5%)
Soft tissue	8 (15.7%)
Urinary	10 (19.6%)
Other	1 (2%)
Uncertain	3 (5.9%)

Of the 51 patients studied, 26 (51%) achieved vasopressor independence at 24 h. Five patients (10%) had died at 28 days. The results of our complexity analysis are detailed in Additional file 1: eTable S1. On principal component analysis of the complexity measures (variables with high loadings are displayed in Additional file 1: eTable 2), the first three components represented 55% of the variance, while PC_1_ represented 26% of variance. Overall the loadings on PC_1_ suggest that higher values of PC_1_ were associated with greater complexity.

### Primary outcome

On Firth logistic regression, PC_1_ was not associated with vasopressor independence (OR 1.04; 95% CI: 0.93–1.16; *p* = 0.54) after controlling for average SBP. The regression results are displayed in Table 3. PC_1_ was also not associated with vasopressor independence (*p*=0.74) after controlling for APACHE II (results of collinearity analysis are presented in Additional file 1: eFigure S1 ). The bivariate model of vasopressor independence had poor calibration (*p*=0.03) by Hosmer-Lemeshow goodness-of-fit (GOF) test and a bootstrapped AUC of 0.61 (IQR: 0.57 to 0.67).

**Table 3:** Results from Firth logistic regressions predicting vasopressor independence

Model	OR (95% CI)	*p*-value
*Primary*		
(Intercept)	10.7 (0.69 to 232.8)	0.09
PC_1_	1.04 (0.93 to 1.16)	0.54
SBP	0.977 (0.95 to 1.00)	0.08
*First ancillary*		
(Intercept)	0.96 (0.56 to 1.65)	0.88
PC_1_	1.02 (0.92 to 1.13)	0.77
*Second ancillary*		
(Intercept)	2.74 (0.53 to 15.8)	0.23
PC_1_	1.02 (0.91 to 1.14)	0.734
APACHEII	0.96 (0.91 to 1.02)	0.19
*Third ancillary*		
(Intercept)	1.72 (0.85 to 3.64)	0.13
PC_1_	0.99 (0.89 to 1.11)	0.88
NEE dose	<0.001 (<0.001 to 0.17)	<0.01

### Secondary outcome

On our prespecified secondary analysis of 28-day mortality, Firth logistic regression suggested a significant association between PC_1_ and 28-day mortality (OR 1.16, 95% CI: 1.01–1.35, *p* = 0.04), as displayed in Table 4. The mortality model was well-calibrated by Hosmer-Lemeshow goodness of fit (*p*>0.20) and had a bootstrapped AUC of 0.87 (95% CI: 0.68 to 0.99). The association of PC_1_ and mortality persisted among each of three ancillary analyses (Table 4): univariate analysis (p=0.016), one controlling for NEE dose (*p*=0.008), and one controlling for APACHE II (*p*=0.053 for PC_1_; APACHE II was not significant, with *p*=0.26).

**Table 4:** Results of Firth logistic regressions predicting 28-day mortality

Model	OR (95% CI)	*p*-value
*Primary*		
(Intercept)	0.020 (<0.001 to 1.48)	0.08
PC1	1.16 (1.01 to 1.35)	0.04
SBP	1.02 (0.974 to 1.06)	0.47
*First ancillary*		
(Intercept)	0.089 (0.024 to 0.225)	*<*0.001
PC1	1.18 (1.03 to 1.38)	0.02
*Second ancillary*		
(Intercept)	0.022 (0.001 to 0.328)	0.004
PC1	1.15 (0.998 to 1.34)	0.05
APACHEII	1.06 (0.962 to 1.17)	0.26
*Third Ancillary*		
(Intercept)	0.048 (0.007 to 0.17)	*<*0.001
PC1	1.22 (1.06 to 1.46)	0.008
NEE dose	174.8 (0.57 to 10.5x105)	0.07

Results of the exact logistic regression, which largely but not entirely corroborated the findings of Firth regression, are displayed in Additional file 1: eTable S3 . In the exploratory analysis of the association between coefficient of variation and PC1, the correlation was not significant (Spearman correlation −0.28, *p* value for linear regression 0.13). Coefficient of variation was also not associated on Firth logistic regression with either vasopressor independence (*p*=0.82) or 28-day mortality (*p*=0.31).

## Discussion

Although we did not demonstrate an association between early SBP complexity and our prespecified primary outcome, vasopressor independence at 24 h, we did observe a possible association between early SBP complexity and our prespecified secondary outcome, 28-day mortality. This association persisted whether controlling for mean systolic blood pressure, vasopressor infusion rate, or admission APACHE II score and was largely robust to an exploratory analysis using exact logistic regression.

Several studies in HRV suggest that increased complexity in HRV is associated with relative health. [14, 17–20] Of note, the large majority of these studies are of the inter-beat interval of heart rate, which is an interval of time related to inputs from all the components of the autonomic nervous system. Two possible sources of complexity exist within SBP: variation in the inter-beat interval (normally measured in HRV), and variations in the amplitude of SBP. In this study, we measured the latter, representing the unique contributions of SBP. We did not control for HRV complexity per se in this research, given the risk of overfitting regression models with a modest number of outcomes. We acknowledge that extreme variation in inter-beat interval could affect the amplitude of SBP, e.g., through variations in stroke volume deriving from differences in filling time. We acknowledge this limitation, which should be explored in future research.

Because many complexity measures are potential candidates for investigation, we felt that the risk of Type 1 statistical error would be prohibitively high for an analysis of all potential complexity measures, especially in light of our modest sample size. We therefore chose, *a priori*, to use the first principal component from PCA. This composite measure, while somewhat more difficult to interpret physiologically, provides an overall measure of complexity. In this case, the first principal component has high loadings for the first standard deviation of a Poincare plot and related measures. This suggests that patients who have substantial and irregular swings in their SBP are at increased risk for mortality. In our cohort, higher complexity was therefore associated with worse outcomes, which is different from what is observed with heart rate variability. It may be that large, irregular changes in SBP are deleterious, but other patterns of complexity might still be beneficial. This novel observation will require further validation in larger cohorts but suggests that the relationships observed in the monitoring of inter-beat intervals may not be applicable to the time series of arterial blood pressure values.

One important aspect of this study is the use of principal component analysis to reduce highly multidimensional data to improve efficiency of analysis. The modern ICU often collects large amounts of physiologic data, laboratory results, imaging, and comorbidities into an electronic record. These data are complex and multidimensional. [21] Discovering subtle relationships between these data and clinical outcomes is difficult using traditional statistical techniques. The main advantage of principal component analysis is that one can use more traditional analysis techniques on the resulting components, while limiting the risk of Type 1 error.

Since substantial variation in systolic blood pressure (or pulse pressure) across a single respiratory cycle may indicate hypovolemia and in sepsis may indicate the early, hypovolemic phase of sepsis, we performed an exploratory analysis. That analysis did not suggest that our findings were primarily due to the identification of patients with persistent hypovolemia.

Strengths of this study include its prospective nature and its focus on early sepsis, using a measurement that can be obtained in a short interval. We acknowledge that this short interval (5 min) may have some downsides as well. We chose this time interval because we felt it was likely to be a clinically relevant time scale. However, we are unable to comment on whether alternative window sizes would yield different results. We are skeptical that substantially longer time windows would be clinically useful (independent of their statistical properties) given how time-sensitive management decisions are early in sepsis. Future work could assess explicitly the tradeoffs involved in choosing different windows for computing SBP variability.

The question of generalizability is important. Notably, although our patients had high severity of illness, the observed mortality was low. This is compatible with longstanding observations at the study hospital that the standardized mortality ratio for APACHE II is consistently < 0.5. Whether this reflects the effects of protocol-based care [22] or differences in the patient population is unknown, but the low mortality may affect generalizability of our findings. Specifically, whether these observations are true among septic patients with higher mortality is thus unknown.

Our study is limited by its modest sample size. Our model therefore runs the risk of overfitting, as few patients died in 28 days, although Firth regression is designed to address this shortcoming. Regardless, our observations will need to be validated in a larger cohort of patients, as the risk of spurious inference is substantial with a modest sample size. We also cannot exclude the possibility of selection bias, as subjects required arterial blood pressure monitoring, but almost one-third of otherwise eligible subjects had arterial tracings that were inadequate for analysis. External validation of the peak identification algorithm employed will also be important for future validations of our technique; the high rate of uninterpretable waveforms may also limit generalizability.

We also acknowledge evolving data about the risks of excessive fluid resuscitation; [23–26] since we did not measure fluid administered in these patients, we cannot control for or comment on that phenomenon.

In summary, we provide early evidence suggesting the possibility that the early complexity of systolic blood pressure in sepsis may be associated with mortality.

## Conclusion

In a prospective observational study, the complexity of systolic blood pressure variation early in the course of sepsis appeared to be associated with 28-day mortality. This apparent effect appears to be independent of preload deficiency during the hypovolemic phase of sepsis. Further research is indicated to determine the factors contributing to this association.

## Additional file

Additional file 1: Online Data Supplement including complexity metrics measured of the data and other SPB variability complexity data. (DOCX 235 kb)

## Abbreviations

APACHE II: Acute physiology and chronic health evaluation II
AUC: Area under the curve
CI: Confidence interval
EMR: Electronic medical record
HF: High frequency
HRV: Heart rate variability
ICU: Intensive care unit
LF: Low frequency
NEE: Norepinephrine
OR: Odds ratio
PC_1_: First principal component
SOFA: Sequential organ failure assessment
VLF: Very low frequency
