# Supplementary material for: Systolic blood pressure variability in patients with early severe sepsis or septic shock: a prospective cohort study
Source: BMC Anesthesiol. 2017 Jun 17;17:82. doi: 10.1186/s12871-017-0377-4 (PMC5473993; doi:10.1186/s12871-017-0377-4)
Supplement: Additional file 1: — Online Data Supplement including complexity metrics measured of the data and other SPB variability complexity data. (DOCX 235 kb) [file 12871_2017_377_MOESM1_ESM.docx]

ONLINE DATA SUPPLEMENT

Systolic blood pressure variability in patients with early severe sepsis or septic shock: a prospective cohort study

Yi Tang, MS^3^ (Yi.Tang@utah.edu), Jeff Sorenson, MStat (Jeff.Sorenson@imail.org), Michael Lanspa, MD^1,2^ (Michael.Lanspa@imail.org), Colin K. Grissom, MD^1,2^ (Colin.Grissom@imail.org), V.J. Mathews, PhD^3^ (Mathews@ece.utah.edu), Samuel M. Brown, MD MS^1,2^ (Samuel.Brown@imail.org)

^1^Pulmonary and Critical Care, Intermountain Medical Center

5121 Cottonwood St, Murray, UT 84107

^2^Pulmonary and Critical Care, University of Utah School of Medicine

30 N 1900 E, Salt Lake City, UT 84132

^3^Electrical and Computer Engineering, University of Utah

50 Central Campus Dr #2110, Salt Lake City, UT 84112

SUPPLEMENTAL METHODS 1

Assessment of collinearity of admission APACHE II and PC_1_ 2

Exploratory exact logistic regression analyses 2

Description of a Poincare plot 2

SUPPLEMENTAL RESULTS 3

eTable 1. Complexity metrics measured 3

eTable 2. Loadings of Complexity Metrics on Principal Component 1 6

Assessment of collinearity of admission APACHE II and PC_1_ 7

eFigure 1: Scatterplot of APACHE II and PC_1_ 8

eTable 3: Results of post hoc sensitivity analyses using exact logistic regression for vasopressor independence and 28-day all-cause mortality. 8

# SUPPLEMENTAL METHODS

## Assessment of collinearity of admission APACHE II and PC_1_

For the purposes of regression modeling, we assessed the collinearity of APACHE II and PC_1_. We did so by fitting relevant scatterplots of fitted regression lines (see eFigure 1). We calculated a variable inflation factor and compared the coefficients of linear and quadratic regression lines.

## Exploratory exact logistic regression analyses

As a *post hoc* analysis, we used exact logistic regression to repeat our primary and secondary analyses. We used the elrm package, which employs Markov Chain Monte Carlo simulation to derive estimates. We report the results of these analyses in eTable 1.

## Description of a Poincare plot

A Poincare plot (also known as a “return map”; see eFigure 1) describes the relationship between subsequent measurements in a series, alternatively its “self-similarity.”[1] By convention, the first standard deviation (SD1) of the Poincare plot represents the degree of dissimilarity of adjacent measurements, while the second standard deviation (SD2) represents the spread of values. In health, SD2 tends to be relatively high (indicating the effect of a normal sinus node arrhythmia), while in disease SD2 tends to be lower (indicating a stress system with low overall variation). SD1 is generally low and tends to be elevated in settings where the normal rhythm functions of the sinus node are disrupted in some way (e.g., by atrial or ventricular ectopic beats).


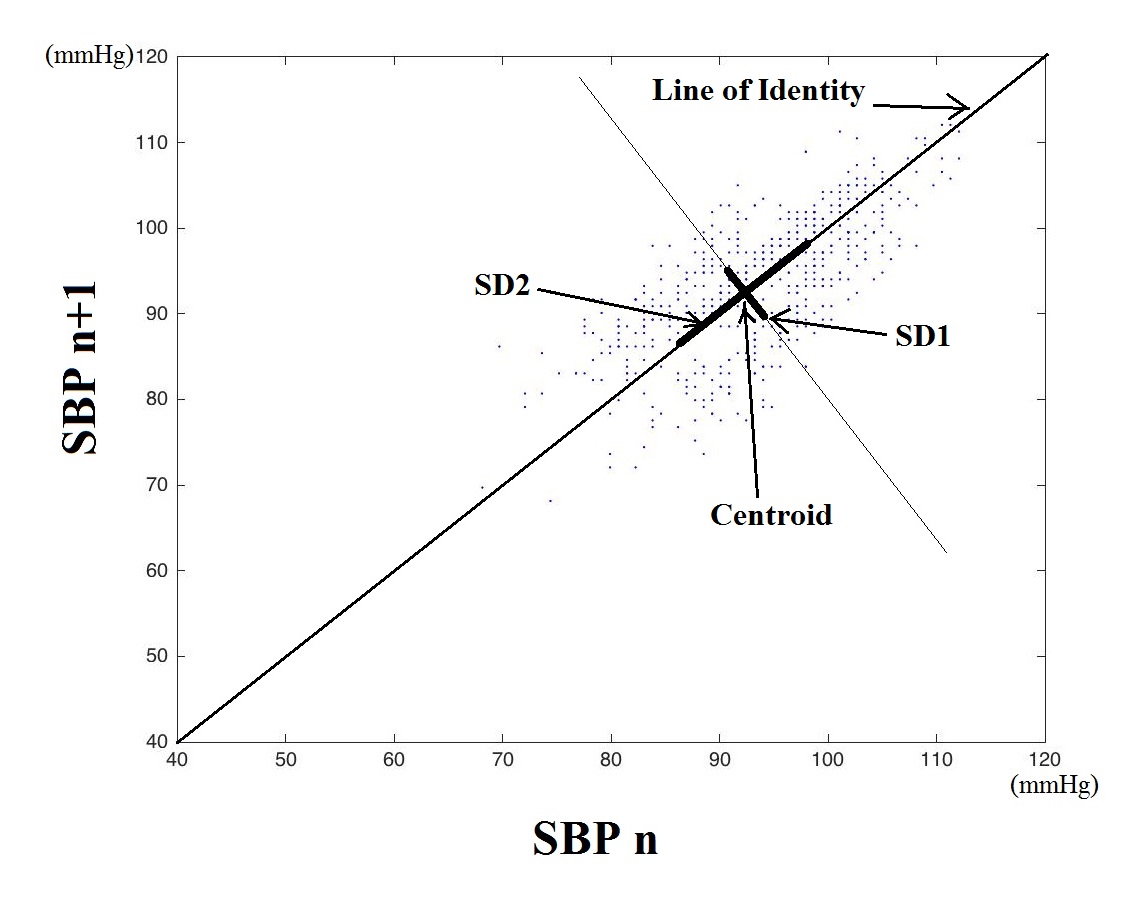


# SUPPLEMENTAL RESULTS

## eTable 1. Complexity metrics measured

| **Complexity measure** | **Median (**IQI**)** |
| --- | --- |
| Mean | 98.3 (86.8 - 108.9) |
| Standard Deviation | 7.9 (6.6 - 11.6) |
| Coefficient of variation | 0.1 (0.1 - 0.1) |
| Poincare SD1 | 5.4 (3.6 - 7.8) |
| Poincare SD2 | 9.9 (8.3 - 14.4) |
| Square root of the mean squared differences of N successive NN intervals | 7.7 (5.1 - 11) |
| Sample entropy | 1.3 (1 - 1.5) |
| LFHF ratio Lomb Scargle | 0.7 (0.4 - 1.3) |
| LF Power Lomb Scargle | 0.1 (0.1 - 0.2) |
| HF Power Lomb Scargle | 0.2 (0.1 - 0.2) |
| Wavelet AUC | 10.2 (8.3 - 13) |
| Detrended Fluctuation Analysis: Alpha1 | 0.8 (0.7 - 1) |
| Detrended Fluctuation Analysis: Alpha2 | 0.8 (0.7 - 1) |
| Detrended Fluctuation Analysis: Alpha | 0.8 (0.7 - 1) |
| Largest Lyapunov exponent | 1.3 (1 - 1.6) |
| Skewness | 0.6 (-0.4 - 4.2) |
| Kurtosis | 15.8 (3.6 - 47.6) |
| Power Law Slope Frequency based | -0.1 (-0.2 - 0) |
| Power Law Y Intercept Frequency based | 9.5 (8.3 - 10.2) |
| Power Law Intercept Frequency based | 52.3 (0 - 142.1) |
| Power Law Goodness of Fit Frequency based | 0.8 (0.8 - 0.9) |
| Power Law Slope Lomb-Scargle | 0 (-0.1 - 0.1) |
| Power Law Y Intercept Lomb-Scargle | 5.8 (5.7 - 6) |
| Power Law Intercept Lomb-Scargle | 25.3 (-49.2 - 75.1) |
| Power Law Goodness of Fit Lomb-Scargle | 0 (0 - 0) |
| Power Law Slope Histogram based | -0.7 (-1.1 - -0.6) |
| Power Law Y Intercept Histogram based | -0.2 (-0.9 - 0.5) |
| Power Law Intercept Histogram based | -0.2 (-1.6 - 0.5) |
| Power Law Goodness of Fit Histogram based | 0.6 (0.4 - 0.7) |
| Detrended Fluctuation Analysis: area under the curve | 1.4 (1.2 - 1.5) |
| Multiscale Entropy | 1.2 (0.9 - 1.6) |
| LFHF ratio | 3.4 (1.1 - 5.2) |
| VLF Power | 4.8e+07 (6.4e+06 - 2.6e+08) |
| LF Power | 7.9e+07 (1.0e+07 - 4.4e+08) |
| HF Power | 3.3e+07 (1.1e+07 - 1.2e+08) |
| VLF Power Lomb-Scargle | 0.3 (0.1 - 0.4) |
| Activity | 9.8e+03 (7.6e+03 - 1.2e+04) |
| Mobility | 0.1 (0 - 0.1) |
| Complexity | 19.6 (15.4 - 27) |
| Embedding scaling exponent | 1 (1 - 1) |
| Recurrence quantification analysis: percentage of recurrences | 2.8 (1.8 - 7.9) |
| Recurrence quantification analysis: percentage of determinism | 22.1 (10 - 34.9) |
| Recurrence quantification analysis: maximum diagonal line | 0 (0 - 0.1) |
| Recurrence quantification analysis: average diagonal line | 0 (0 - 0) |
| Recurrence quantification analysis: Shannon entropy of the diagonals | 1.6 (1.2 - 2) |
| Recurrence quantification analysis: determinism/recurrences | 4.8 (2.4 - 7.2) |
| Recurrence quantification analysis: percentage of laminarity | 0.7 (0 - 7.6) |
| Recurrence quantification analysis: maximum vertical line | 0 (0 - 0) |
| Recurrence quantification analysis: trapping time | 0 (0 - 0) |
| Recurrence quantification analysis: Shannon entropy of the vertical lines | 0 (0 - 1.4) |
| Shannon entropy | 4.7 (4.4 - 5.3) |
| Plotkin and Swamy energy operator average energy | 19.4 (1.9 - 28.7) |
| Teager energy operator average energy | 37.7 (17 - 79.2) |
| Symbolic dynamics: percentage of 0 variations sequences, uniform case | 0.6 (0.3 - 0.8) |
| Symbolic dynamics: percentage of 1 variations sequences, uniform case | 0.3 (0.2 - 0.4) |
| Symbolic dynamics: percentage of 2 variations sequences, uniform case | 0.1 (0 - 0.2) |
| Symbolic dynamics: percentage of 0 variations sequences, non-uniform case | 0.4 (0.3 - 0.5) |
| Symbolic dynamics: percentage of 1 variations sequences, non-uniform case | 0.4 (0.4 - 0.5) |
| Symbolic dynamics: percentage of 2 variations sequences, non-uniform case | 0.2 (0.1 - 0.2) |
| Symbolic dynamics: forbidden words, uniform case | 192 (171.5 - 201) |
| Symbolic dynamics: forbidden words, non-uniform case | 31 (22 - 38) |
| Symbolic dynamics: Shannon entropy, uniform case | 2.7 (1.8 - 4.3) |
| Symbolic dynamics: Shannon entropy, non-uniform case | 4 (3.5 - 4.5) |
| Symbolic dynamics: modified conditional entropy, uniform case | 1 (0.8 - 1.2) |
| Symbolic dynamics: modified conditional entropy, non-uniform case | 1.3 (1.2 - 1.5) |
| Form factor | 1.6 (1.4 - 1.9) |
| Fuzzy entropy | 2.8 (2.3 - 3.1) |
| Grid transformation feature: grid count | 2.3 (1.5 - 3) |
| Rescaled detrended range analysis | 0.9 (0.8 - 1) |
| Higuchi scaling exponent | 1.9 (1.8 - 2) |
| Allan factor distance from a Poisson distribution | -0.3 (-0.3 - -0.3) |
| Fano factor distance from a Poisson distribution | -0.4 (-0.5 - -0.4) |
| Index of variability distance from a Poisson distribution | -0.3 (-0.6 - -0.1) |
| Scaled windowed variance | 0.2 (0.1 - 0.3) |
| Kullback-Leibler permutation entropy | 0 (0 - 0) |
| Multiscale time irreversibility asymmetry index | 0 (-0.1 - 0) |
| Lee parameter | 1.2 (1.1 - 1.3) |
| Poincaré plot cardiac sympathetic index | 1.9 (1.5 - 2.3) |
| Poincaré plot cardiac vagal index | 2.9 (2.7 - 3.2) |
| Mean of the differences | 0 (0 - 0) |
| Standard deviation of the differences | 7.7 (5.1 - 11) |
| Interquartile range | 7.8 (5.5 - 9.8) |
| Predictive feature: error from an Autoregressive model | 5.7 (3.7 - 8.3) |
| Multifractal spectrum cumulant of the first order | 0.5 (0.4 - 0.6) |
| Multifractal spectrum cumulant of the second order | -0.1 (-0.2 - 0) |
| Multifractal spectrum cumulant of the third order | 0 (0 - 0.1) |
| Kolmogorov-Sinai entropy | 0 (0 - 0.1) |
| Scale dependent Lyapunov exponent slope | -7.5 (-8.5 - -6.7) |
| Scale dependent Lyapunov exponent mean value | 5.6 (4.3 - 6.9) |
| Dynamical moment of the second order | 1.4 (-1.4 - 7.7) |
| Dynamical moment of the third order along the principal bisector | 9.2 (-28.5 - 78.9) |
| Dynamical moment of the third order along the secondary bisector | 159 (-117.2 - 4364.7) |
| Dynamical moment of the third order along the x axes | 254.9 (-37.9 - 2866.8) |
| Dynamical moment of the third order along the y axes | 15.2 (-99.7 - 2704.8) |
| Diffusion entropy | 0.1 (0 - 0.2) |
| IQI: inter-quartile interval |  |

## eTable 2. Loadings of Complexity Metrics on Principal Component 1

| **Complexity Metric** | **Loading on PC1*** |
| --- | --- |
| Predictive feature: error from an Autoregressive model | 0.945 |
| Poincaré SD1 | 0.930 |
| Standard deviation of the differences | 0.930 |
| Square root of the mean squared differences of N successive NN intervals | 0.930 |
| Standard Deviation | 0.927 |
| Mobility | 0.922 |
| Teager energy operator average energy | 0.916 |
| Poincaré plot cardiac vagal index | 0.907 |
| Wavelet area under the curve | 0.897 |
| Symbolic dynamics: forbidden words, non-uniform case | -0.895 |
| Coefficient of variation | 0.892 |
| Lee parameter | 0.883 |
| Fuzzy entropy | 0.873 |
| Poincaré SD2 | 0.863 |
| Symbolic dynamics: Shannon entropy, non-uniform case | 0.847 |
| Correlation dimension | 0.840 |
| Detrended fluctuation analysis: area under the curve | 0.801 |
| Symbolic dynamics: modified conditional entropy, non-uniform case | 0.801 |
| HF Power | 0.781 |
| HF Power Lomb-Scargle | 0.763 |
| Detrended fluctuation analysis: Alpha 2 | -0.723 |
| Symbolic dynamics: percentage of 2 variations sequences, non-uniform case | 0.721 |
| Interquartile range | 0.703 |
| Complexity | -0.673 |
| VLF Power Lomb-Scargle | -0.666 |
| Symbolic dynamics: percentage of 0 variations sequences, non-uniform case | -0.658 |
| Correlation Dimension: Local Exponents | 0.654 |
| Embedding scaling exponent | -0.653 |
| Detrended fluctuation analysis: overall alpha | -0.630 |
| Allan factor distance from a Poisson distribution | 0.610 |
| Rescaled detrended range analysis | -0.601 |
| Kolmogorov-Sinai entropy | 0.587 |
| Power Law Y Intercept Frequency based | 0.569 |
| Higuchi scaling exponent | 0.525 |
| *Restricted to variables with loadings with an absolute value > 0.5  PC1: first principal component from principal components analysis | |

## Assessment of collinearity of admission APACHE II and PC_1_

The variance inflation factor (VIF) was approximately one, and the coefficients of the linear and quadratic regressions were not significantly different (eFigure 1). We therefore concluded that APACHE II and PC_1_ were collinear.

## eFigure 1: Scatterplot of APACHE II and PC_1_


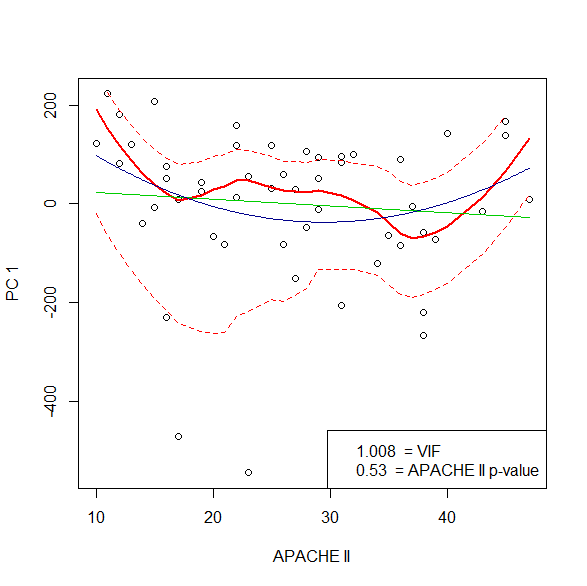


The green curve represents the bivariate regression equation (p_apache_ = 0.53); the blue curve represents the quadratic regression equation (p_apache_ = 0.08, p_apache−squared_ = 0.09), and the red curve is the LOESS smoothing curve.

## eTable 3: Results of post hoc sensitivity analyses using exact logistic regression for vasopressor independence and 28-day all-cause mortality.

| Outcome | Model | OR_PC1_ | 95% CI_PC1_ |
| --- | --- | --- | --- |
| Vasopressor independence | Primary | 1.14 | (0.89 to 1.66) |
| Vasopressor independence | First ancillary | 1.02 | (0.93 to 1.13) |
| Vasopressor independence | Second ancillary | 1.02 | (0.91 to 1.14) |
| Vasopressor independence | Third ancillary | 0.99 | (0.89 to 1.11) |
| 28-day mortality | Primary | 1.21 | (0.85 to 4.62) |
| 28-day mortality | First ancillary | 1.26 | (1.05 to 1.79) |
| 28-day mortality | Second ancillary | 0.83 | (0.42 to 1.30) |
| 28-day mortality | Third ancillary | 1.30 | (1.01 to 1.97) |

First ancillary analysis: univariate analysis of PC_1_ vs. outcome; second ancillary analysis: bivariate analysis of PC_1_ and APACHE II vs. outcome; third ancillary analysis: bivariate analysis of PC_1_ and vasopressor dose (in norepinephrine equivalent doses).

Analyses in blue font differed (in terms of whether the confidence interval included unity) from the prespecified analyses that employed Firth logistic regression.

## References

1. Huikuri HV, Seppanen T, Koistinen MJ, Airaksinen J, Ikaheimo MJ, Castellanos A, Myerburg RJ: **Abnormalities in beat-to-beat dynamics of heart rate before the spontaneous onset of life-threatening ventricular tachyarrhythmias in patients with prior myocardial infarction.** *Circulation* 1996, **93:**1836-1844.
